# In-Depth Study on the Application of a Graphene Platelet-reinforced Composite to Wind Turbine Blades

**DOI:** 10.3390/ma17163907

**Published:** 2024-08-07

**Authors:** Hyeong Jin Kim, Jin-Rae Cho

**Affiliations:** 1 Department of Mechanical Engineering, University College London, London WC1E 7JE, UK; hj.kim.22@ucl.ac.uk; 2 Department of Naval Architecture and Ocean Engineering, Hongik University, Sejong 30016, Republic of Korea

**Keywords:** wind turbine blade, graphene platelet (GPL), mechanical characteristics, cost–benefit analysis, finite element structural analysis

## Abstract

Graphene platelets (GPLs) are gaining popularity across various sectors for enhancing the strength and reducing the weight of structures, thanks to their outstanding mechanical characteristics and low manufacturing cost. Among many engineering structures, wind turbine blades are a prime candidate for the integration of such advanced nanofillers, offering potential improvements in the efficiency of energy generation and reductions in the construction costs of support structures. This study aims to explore the potential of GPLs for use in wind turbine blades by evaluating their impact on material costs as well as mechanical performance. A series of finite element analyses (FEAs) were conducted to examine the variations of mechanical performances—specifically, free vibration, bending, torsional deformation, and weight reductions relative to conventional fiberglass-based blades. Details of computational modeling techniques are presented in this paper. Based on the outcomes of these analyses, the mechanical performances of GPL-reinforced wind turbine blades are reviewed along with a cost–benefit analysis, exemplified through a 5MW-class wind turbine blade. The findings affirm the practicality and benefits of employing GPLs in the design and fabrication of wind turbine blades.

## 1. Introduction

Since their discovery, graphene platelets (GPLs) have attracted interest as innovative nanofillers that can reinforce composites owing to their excellent physical, mechanical, and thermal characteristics. GPLs, recognized as high-strength and ultra-light nanomaterials, possess material properties comparable to those of carbon nanotubes (CNTs). However, the production and sales costs of GPLs are lower than CNTs. Due to their larger surface area compared to CNTs, GPLs facilitate more flexible interactions and load transfer within a matrix [1]. Therefore, over the past decade, GPL-reinforced composites (GPLRCs) have garnered more interest than CNT-reinforced composites (CNTRCs) [2].

Improvement in the mechanical performance of structures with the superior material properties of GPLs has been proven in many studies. The following are representative cases. Rafiee et al. [3] theoretically and experimentally demonstrated that epoxy composites reinforced with 0.1 wt.% GPLs are superior to single-walled carbon nanotubes (SWCNTs) and multi-walled carbon nanotubes (MWCNTs) of the same weight fraction and pure epoxy materials with respect to mechanical characteristics, such as strength, stiffness, and fracture toughness. Rafiee et al. [4] demonstrated that reinforcing a beam with 0.1 wt.% GPLs increases its buckling strength by 52% compared to pure epoxy materials. Additionally, the critical buckling strength is increased by 43% and 32% compared to beams reinforced with SWCNTs and MWCNTs of the same weight, respectively. Parashar and Mertiny [5] assessed the buckling strength of GPL-reinforced plates using finite element analysis (FEA). They found that plates with a 6% volume fraction of GPLs exhibited a 26% increase in buckling strength compared to plates without reinforcement. Feng et al. [6] noted that epoxy beams reinforced with GPLs showed a substantial decrease in deflection compared to those without any reinforcement, with the most notable enhancement in bending performance occurring when GPLs were used to reinforce both the upper and lower sections of the beams. Gholami and Ansari [7] studied the large deflection behavior of square plates reinforced with GPLRCs, using a functionally graded distribution. They found that as the GPL content increased, the stiffness of the plate also increased, leading to a significant reduction in maximum deflection. In addition, various research cases that improved the performance of structures using GPLRCs have been systematically summarized in review papers [8,9,10].

Owing to the recent surge in extreme weather events caused by the use of fossil fuels, global efforts have intensified to combat climate change (e.g., the Paris Agreement [11] and regulations on greenhouse gas (GHG) emissions from ships [12]). The importance of renewable energy, such as wind energy, as a countermeasure has been emphasized. In tandem with this global trend, interest in the application of cutting-edge materials to wind turbines is increasing. For wind turbine blades, fiberglass composites have been used as the main materials. However, in recent years, materials such as basalt–carbon hybrid fibers [13,14], SiO_2_ and Al_2_O_3_ [15], bamboo [16,17], and carbon fibers [18,19] have been investigated to replace conventional fiberglass composites.

Recently, research has also focused on the application of nanocomposites. For example, Boncel et al. [20] examined the use of CNTs in wind turbine blades, evaluating both their mechanical performance and economic feasibility beyond the current laboratory research stage. Buyuknalcaci et al. [21] also examined the applicability of CNTs to wind turbine blades. In particular, they expected that the service life of wind turbine blades would be significantly extended by eliminating fatigue cracks, which are one of the main causes of damage to wind turbine blades. This demonstrates that research results on the use of nanocomposites in wind turbine blades have been continuously reported. However, the use of GPLs in wind turbine blades has not been reported despite their easy mass production, relatively low production cost, and their application to metals, concrete, and electronic equipment [22,23,24]. Therefore, in the present study, the mechanical characteristics and economic feasibility of wind blades incorporating GPLs were analyzed to evaluate the potential application of GPLs in future wind turbine blades.

In this regard, a sophisticated analysis model was developed based on the SNL 61.5 m model, which is a 5MW-class wind turbine blade model [25]. The mechanical characteristics of GPL-reinforced wind turbine blades were then analyzed using the finite element method. The validity of the developed analysis model was confirmed by comparing its results with those found in the existing literature. The mechanical performances (e.g., natural frequency, bending, and torsion) of wind turbine blades were precisely analyzed based on the GPL content and reinforcement position using the developed analysis model. In addition, economic feasibility was evaluated by analyzing the weight change and material cost of the blades with the GPLs applied. In conclusion, the applicability of GPLs to wind turbine blades as a future material was assessed by combining the results obtained through this study.

## 2. Wind Turbine Blade Reinforced with GPLs

### 2.1. Effective Material Properties of GPLRCs

In the present study, the effective material properties were determined by combining the material properties of GPLs as nanofillers and epoxy as the matrix. Traditionally, GPLRCs have been modeled as a homogeneous and isotropic material because GPLs act as an effective rectangular solid [26,27,28,29]. To facilitate a straightforward comparison of the mechanical performance of GPL-reinforced wind turbine blades and those made with traditional fiberglass composites, the GPLs were assumed to be uniformly distributed throughout the volume of the GPLRC. Table 1 indicates the material properties of GPL and epoxy. The effective material properties of Poisson’s ratio ν and density ρ were determined based on the linear rule of mixtures using Equations (1) and (2).

(1) $$\nu_{eff}=V_{GPL}\;\nu_{GPL}+V_{m}\;\nu_{m}$$

(2) $$\rho_{eff}=V_{GPL}\;\rho_{GPL}+V_{m}\;\rho_{m}$$

where V represents the volume fraction of the material. The subscripts eff, GPL, and m correspond to the effective material property, *GPL*, and matrix (i.e., epoxy), respectively.

The effective elastic modulus of the GPLRC, denoted as $E_{eff}$, was calculated using the Halpin–Tsai micromechanical modeling approach, as described in Equations (3) and (4) below. Here L, T represent the longitudinal and transverse directions, while GPL and m stand for graphene platelet and matrix, respectively.

(3) $$E_{eff}=\frac{3}{8}\cdot \frac{1+\xi_{L}\eta_{L}V_{GPL}}{1-\eta_{L}V_{GPL}}E_{m}+\frac{5}{8}\cdot \frac{1+\xi_{T}\eta_{T}V_{GPL}}{1-\eta_{T}V_{GPL}}E_{m}$$

(4) $$\eta_{L}=\frac{E_{GPL}-E_{m}}{E_{GPL}+\xi_{L}E_{m}},\;\eta_{T}=\frac{E_{GPL}-E_{m}}{E_{GPL}+\xi_{T}E_{m}}$$

with $\xi_{L}=2l_{GPL}/t_{GPL}$ and $\xi_{t}=2w_{GPL}/t_{GPL}$. Here, the length, width, and thickness of the GPLs were set as $l_{GPL}=2.5\;\mu m$, $w_{GPL}=1.5\;\mu m$, and $t_{GPL}=1.5\;\mu m$ by referring to the values presented in the work of Rafiee et al. [16]. Table 2 represents examples of the effective material properties of the GPLRC, calculated using the abovementioned material modeling method.

### 2.2. Finite Element Model of GPL-Reinforced Wind Blade

**Figure 1:**
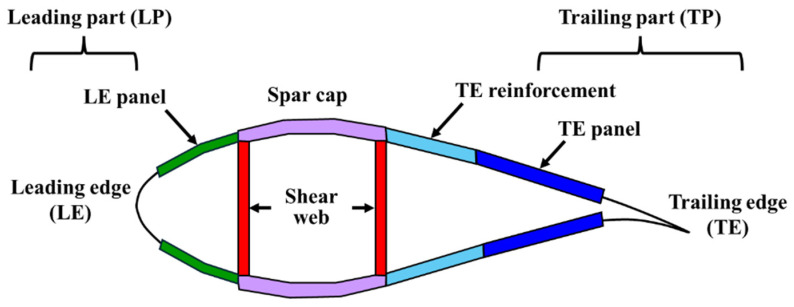
Cross-section of wind turbine blade.

The finite element model of a GPL-reinforced wind blade closely resembles that of traditional fiberglass-composite-based wind blade, except for the difference in the material. In this context, this section describes the finite element model of a fiberglass composite-based wind blade which was referred to as the 5MW-class SNL 61.5 m model. This model was chosen because numerous references in the literature have adopted 5MW-class wind turbine blades, providing many reference values for validation. In addition, the SNL 61.5 m model proposed by Resor [25] provides the detailed information necessary for finite element modeling. The airfoil and geometry information of the SNL 61.5 m model is presented in Table A1. More detailed model information is described in the report by Resor [25]. As shown in Figure 1, the cross-section of the wind turbine blade was composed of structures such as the leading edge (LE), LE panel, spar cap, trailing edge (TE), TE reinforcement, and TE panel. Depending on the structure and location in the blade length (span) direction, the materials and their thicknesses change, including Gelcoat, E-LT-5500 (UD), Saertex (DB), SNL (Triax), foam, and carbon (UD). Table A2 summarizes their material properties, whereas Table A3 and Table A4 show the layout of the materials within the blade model.

### 2.3. Loading and Boundary Conditions

**Figure 2:**
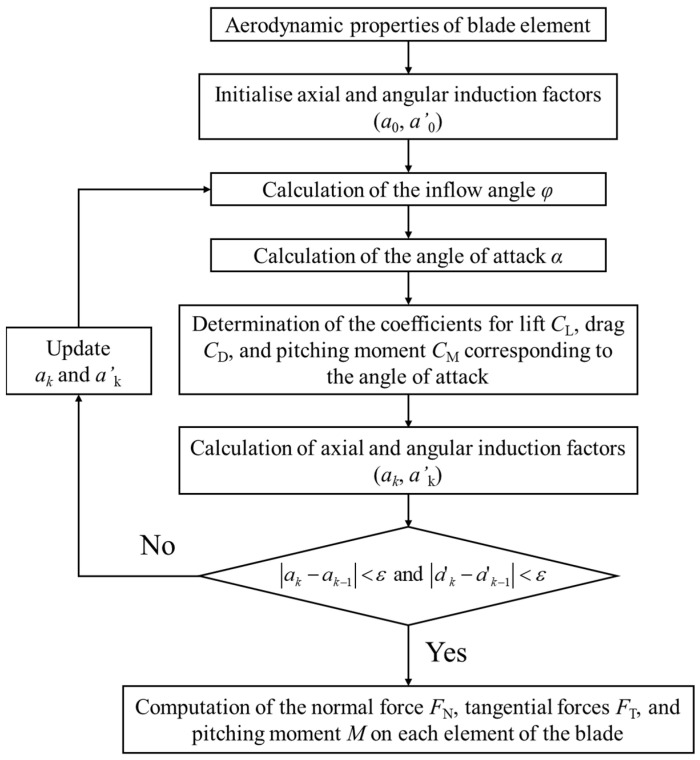
Aerodynamic load calculation process using BEMT.

A wind turbine blade is affected by various loads, such as aerodynamic, inertia, and gravitational loads. Among these, aerodynamic loads are the primary cause of the blades bending and torsional deformation. Therefore, to accurately analyze the blades’ mechanical characteristics, it is necessary to perform an FEA that realistically reflects these aerodynamic loads. The aerodynamic loads acting on the wind turbine blade are primarily calculated using computational fluid dynamics (CFDs) and blade element momentum theory (BEMT) [31]. Due to the high cost and extensive modeling and analysis time required for CFDs, the aerodynamic load calculation method based on BEMT has been widely adopted as a practical approach [31,32,33,34]. In this study, the aerodynamic loads on the 5MW-class wind turbine blades at a rated wind speed of 11.4 m/s were calculated according to the process shown in Figure 2. The details of the aerodynamic load calculation process are summarized in Appendix B.

**Figure 3:**
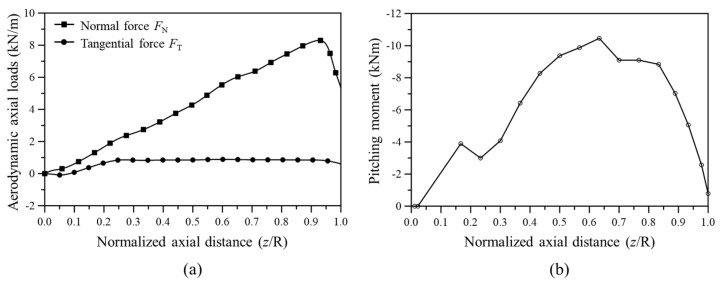
Aerodynamic load distributions along the blade length (span); (**a**) normal and tangential forces, (**b**) pitching moment.

**Figure 4:**
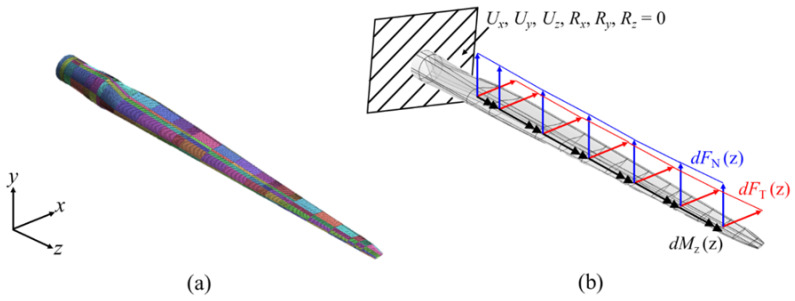
The SNL 61.5 m blade; (**a**) 3-D FEA model, (**b**) loading and boundary conditions.

Figure 3 shows the normal and tangential forces and the pitching moment determined using BEMT. Meanwhile, the blade root was fixed as a boundary condition, as depicted in Figure 4 which shows the FEA model and the loading and boundary conditions.

## 3. Results and Discussion

### 3.1. Validation of the FEA Model

The analysis model used in this study was created using midas-NFX 2024R1, a widely used commercial software for FEA. The composite shell element in midas-NFX was utilized to create the composite laminate within the wind turbine blade, and the element size was set to 80 mm × 80 mm, as referenced from the finite element model in Resor’s report [14]. Table 3 compares the mass of each material used in the analysis models of this study and a previous study. The weights of individual materials and the total weight of the analysis model are comparable to the values presented in the previous study.

Table 4 presents a comparison of the natural frequencies of the SNL 61.5 m blade model, as determined in this study and those reported in previous studies. Each analysis model had similar natural frequencies, and the same mode shape was observed as the mode order increased. This consistency verifies the reliability of the analysis model and the free vibration analysis conducted in this study.

**Figure 5:**
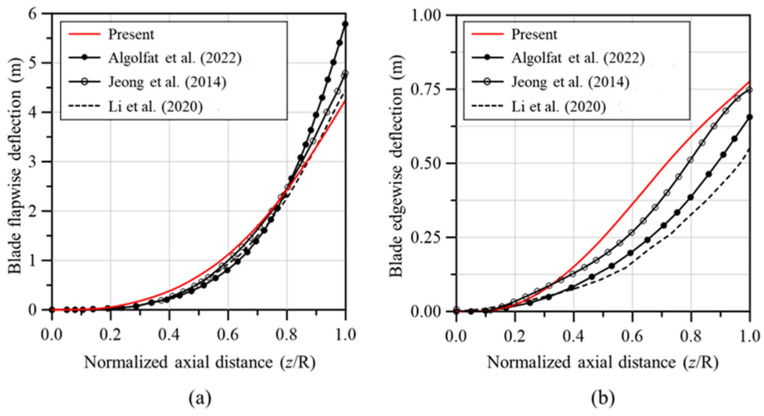
Comparison of blade deflections produced by the aerodynamic loads; (**a**) flapwise deflection, (**b**) edgewise deflection. Algolfat et al. [32]; Jeong et al. [39]; Li et al. [40].

**Figure 6:**
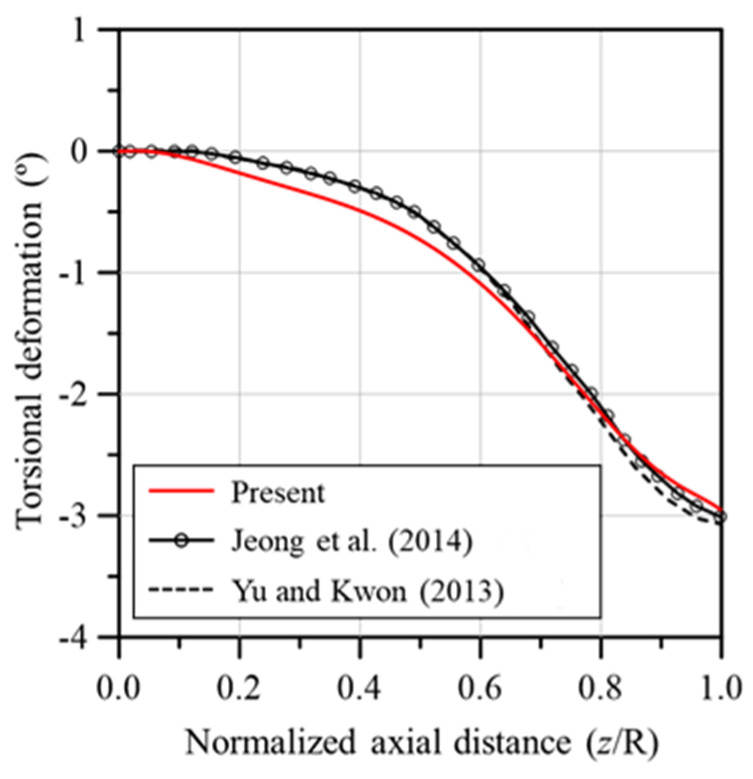
Comparison of blade torsional deformations produced by aerodynamic loads. Jeong et al. [39]; Yu and Kwon [41].

Figure 5 shows a comparison of the deflection in the flapwise and edgewise directions between the analysis models used in this study and those from prior research. The results of this study were consistent with previous studies for the flapwise direction, but the deflection in the edgewise direction differed significantly depending on the literature. The edgewise deflection of the analysis model of this study was higher than the results of other studies, but the maximum deflection of the blade tip was similar to the result of reference [38]. In addition, Figure 6 presents a comparison of the torsional deformation observed in the analysis models from this study and those from previous studies. The torsional deformation was similar for all models. The results of Figure 5 and Figure 6 verified the reliability of the developed analysis model. Because these results are from the analysis that reflected the influence of the aerodynamic loads calculated using BEMT, we can conclude that the accuracy of the aerodynamic loads determined in this study has been verified.

### 3.2. Mechanical Behaviors of GPL-Reinforced Wind Turbine Blade

This section analyzes the mechanical performances of GPL-reinforced wind blades, including the deflection, torsion, stress, and natural frequency. The analysis model was created using GPLRCs of the same thickness as conventional fiberglass composites, i.e., E-LT-5500 (UD), Saertex (DB), and SNL (Triax). An FEA was conducted while varying the volume fraction of the GPL, $V_{CNT}^{*}$. In addition, a numerical analysis was performed by varying the GPL reinforcement position inside the wind turbine blade, and changes in mechanical characteristics due to the GPL reinforcement position were closely analyzed.

#### 3.2.1. Static Bending

**Figure 7:**
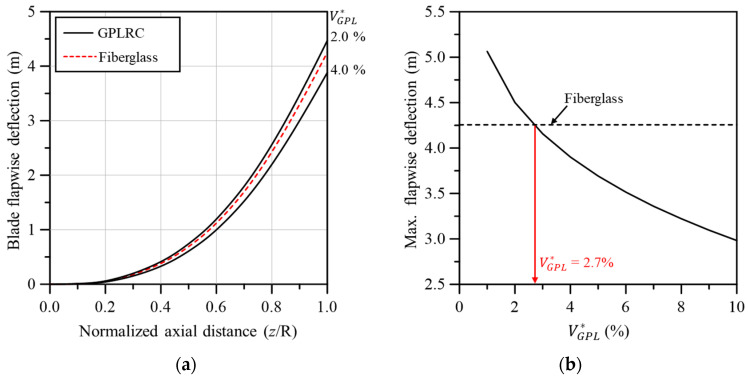
Comparison of flapwise deflection between fiberglass and GPLRC; (**a**) flapwise deflection along the blade span, (**b**) maximum flapwise deflection.

Figure 7 compares the flapwise deflection between the wind turbine blades based on conventional fiberglass composites and the GPL-reinforced blade. As shown in Figure 7a, a similar behavior to the fiberglass-based blade was observed when the volume fraction of GPL was between 2.0% and 4.0%. The more precise volume fraction of the GPL is shown in 7b. The maximum flapwise deflection at the blade tip was comparable to that of the fiberglass-based blade when $V_{GPL}^{*}$ was 2.7%.

**Figure 8:**
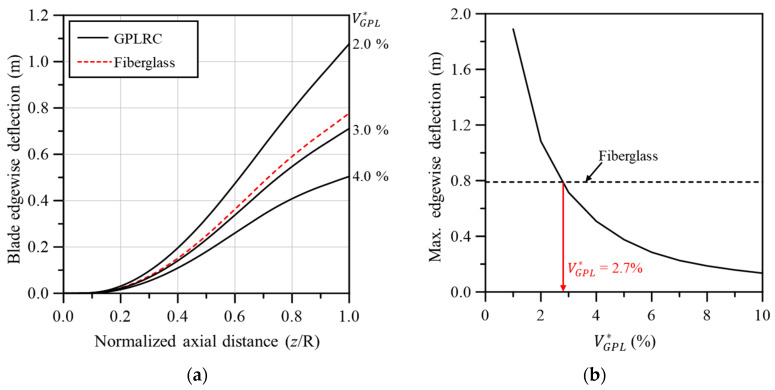
Comparison of edgewise deflection between fiberglass and GPLRC; (**a**) edgewise deflection along the blade span, (**b**) maximum edgewise deflection.

Figure 8 compares the edgewise deflection between the wind turbine blades based on conventional fiberglass composites and the GPL-reinforced blade. Figure 8a shows that similar behavior to the fiberglass-based blade was obtained when $V_{GPL}^{*}$ was between 2.0% and 3.0%. The edgewise deflection appeared to be considerably more sensitive to changes in $V_{GPL}^{*}$ compared to the flapwise deflection shown in Figure 7a. Although the absolute deflection amounts for the flapwise and edgewise deflections were similar, the edgewise deflection appeared relatively more sensitive to changes in $V_{GPL}^{*}$ because its deflection was smaller. As depicted in Figure 8b, the maximum edgewise deflection at the blade tip was comparable to that of the fiberglass-based blade when $V_{GPL}^{*}$ is 2.7%, consistent with the flapwise deflection.

**Figure 9:**
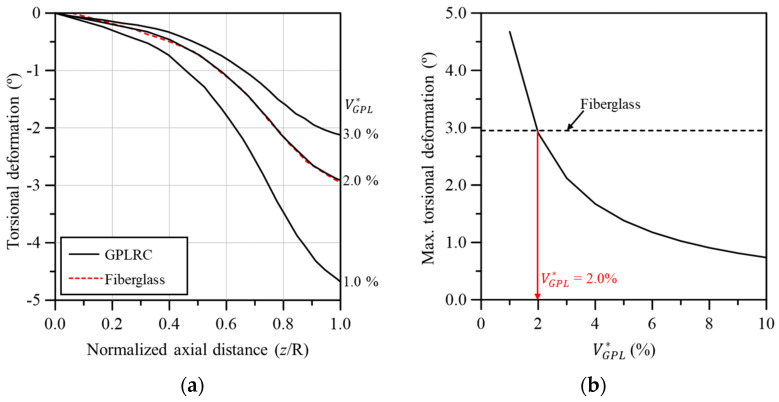
Comparison of torsional deformation between fiberglass and GPLRC; (**a**) torsional deformation along the blade span, (**b**) maximum torsional deformation.

**Figure 10:**
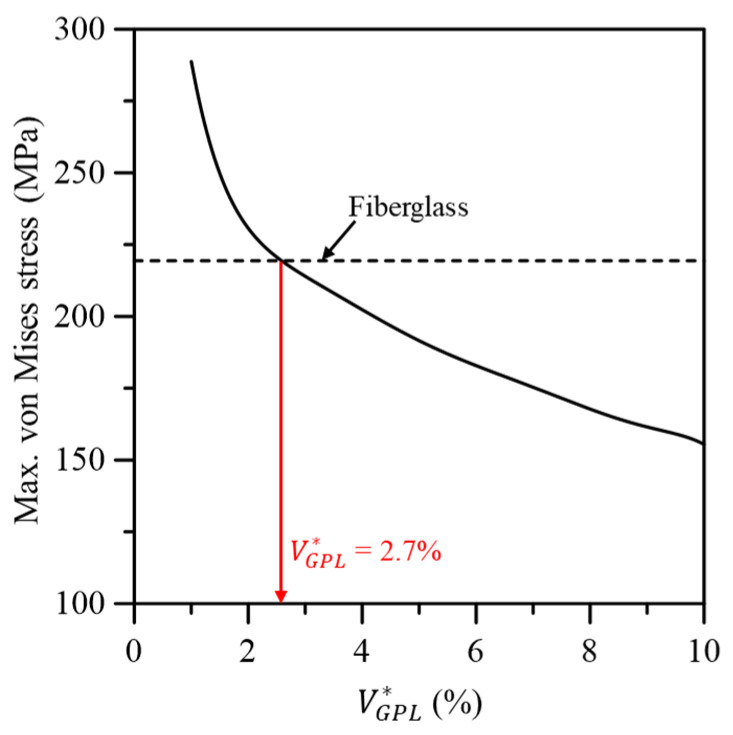
Variation in the maximum von Mises stress to the GPL volume fraction.

Figure 9 compares the torsional deformation between the wind turbine blades based on conventional fiberglass composites and the GPL-reinforced blade. As shown in Figure 9a, similar torsional deformation behavior to the fiberglass-based blade was observed when $V_{GPL}^{*}$ was 2.0%. The same result can also be observed in Figure 9b, confirming that the maximum torsional deformation was comparable to that of the fiberglass-based blade when $V_{GPL}^{*}$ was 2.0%. Figure 10 shows the maximum von Mises stress in the wind turbine blade according to $V_{GPL}^{*}$. For the maximum von Mises stress, a performance similar to the fiberglass-based blade occurred when $V_{GPL}^{*}$ was 2.7% as with the flapwise and edgewise deflections.

**Figure 11:**
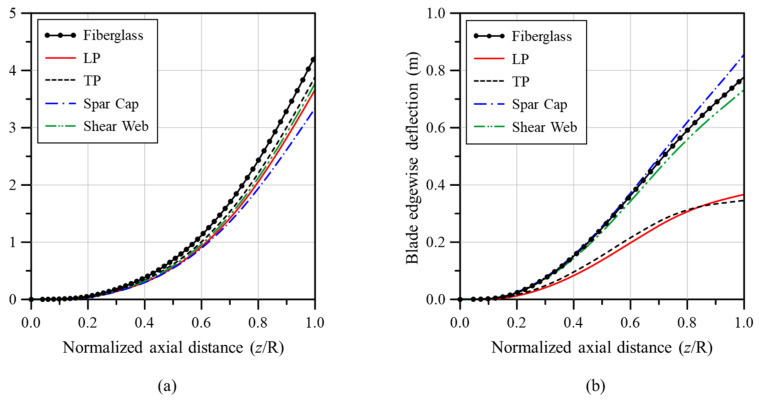

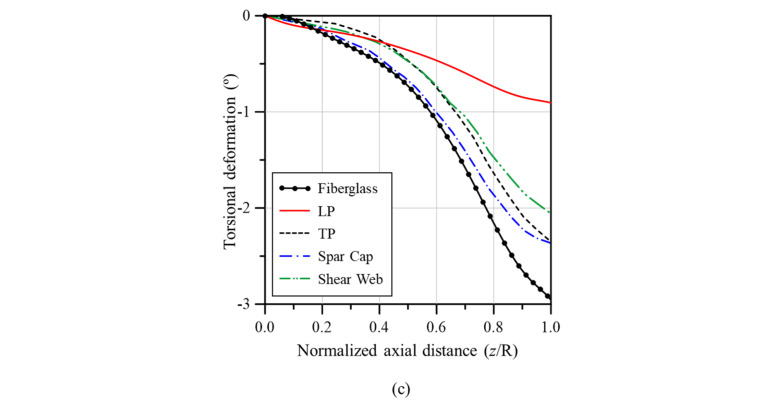
Structural response variations of the wind turbine blade model along the blade span for different reinforcement parts; (**a**) flapwise deflection, (**b**) edgewise deflection, (**c**) torsional deformation.

Next, the flapwise deflection, edgewise deflection, and torsional deformation were compared in Figure 11 according to the GPL reinforcement position inside the wind turbine blade (LP, TP, spar cap, and shear web). Here, the GPL content was 152.3 kg, and the same value was applied to each reinforcement part. This was the mass at $V_{GPL}^{*}=2.7\%$, which exhibited the most similar mechanical performances to the fiberglass-based wind blade in the analysis results presented above. Because the volume depended on the reinforcement part, the same amount of GPLs was used regardless of the area for a fair comparison. First, as shown in Figure 11a, fiberglass showed the largest flapwise deflection, followed by the TP, shear web, LP, and spar cap. All the cases reinforced with GPL exhibited lower flapwise deflection than the fiberglass-based wind turbine blade. Figure 11b presents a comparison of edgewise deflections, with the spar cap showing the largest deflection, followed by the fiberglass, shear web, LP, and TP. Notably, a significant reinforcement effect was observed when the LP and TP were reinforced with GPLs.

Finally, Figure 11c compares the torsional deformation. The fiberglass exhibited the largest deformation, followed by the spar cap, TP, shear web, and LP. The largest reinforcement effect was observed when LP was reinforced with GPLs. Reinforcing the LP consistently demonstrated superior performance, often showing the smallest and second smallest deformations among the different reinforcement positions. By contrast, the other reinforcement positions showed inconsistent performance results. Overall, the results in Figure 11 indicate that reinforcing the LP with GPL is more effective than reinforcing other positions in terms of reducing the flapwise, edgewise, and torsional deformations.

#### 3.2.2. Free Vibration

**Figure 12:**
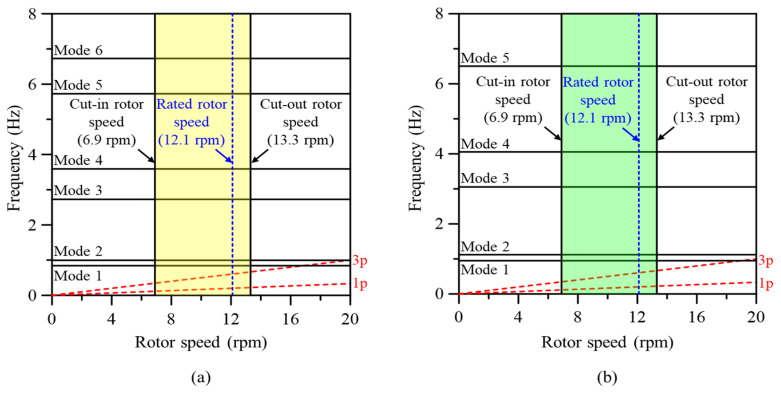
Campbell diagrams of the SNL 61.5 m wind turbine blade; (**a**) with fiberglass, (**b**) with GPLRC ($V_{GPL}^{*}=2.7\%$).

Generally, vibration characteristics must be evaluated in advance to ensure the safety of a structure and prevent accidents. Therefore, the natural frequency characteristics of the wind turbine blade reinforced with GPLs and the fiberglass-based blade were compared to evaluate the safety of GPL-reinforced wind turbine blades. Figure 12 shows the Campbell diagrams of the blades with fiberglass and the GPLRC. The Campbell diagram is an indicator of the resonance of a rotating structure. For a three-bladed wind turbine, we examined whether the 1p and 3p frequencies were consistent with the natural frequency within the operating rotor speed range. As shown in Figure 12, the possibility of resonance was not observed in blades with fiberglass or the GPLRC. However, Figure 12b shows that the difference from the 3p frequency further increased as the natural frequency of the blade increased for the blade with the GPLRC. In conclusion, a wind turbine blade with a GPLRC is safer those that with fiberglass in terms of resonance.

**Figure 13:**
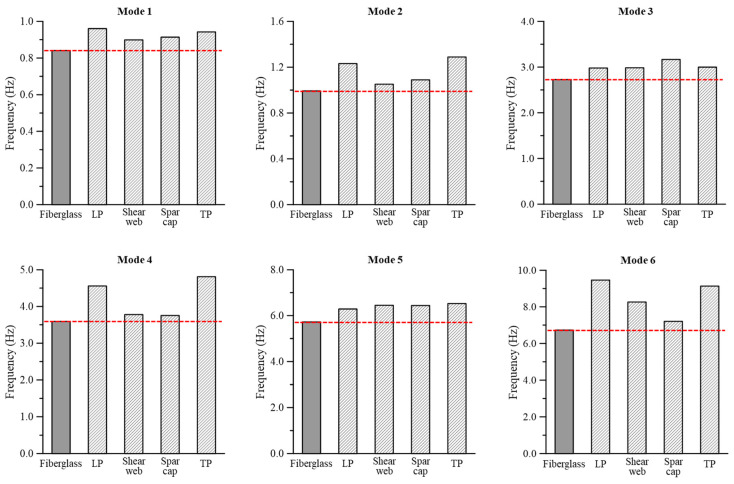
Natural frequencies of the wind turbine blade model for different reinforcement parts.

Next, the natural frequencies up to mode 6 were compared according to the GPL reinforcement position inside the wind turbine blade (LP, TP, spar cap, and shear web) (Figure 13). As in the analysis described in the previous section, the same GPL content (152.3 kg) was applied to each reinforcement part. The results showed that the cases with GPLRCs had higher natural frequencies than those with fiberglass at all mode orders. This tendency appeared to be owed to the relatively high stiffness and low mass of the GPLRC, and it was in good agreement with the well-known natural frequency characteristics of GPL-reinforced composites.

Meanwhile, the difference in natural frequency among the reinforcement parts was relatively small at 1, 3, and 5 mode orders that involved the flapwise mode shape. In contrast, the differences at 2, 4, and 6 mode orders that involved the edgewise and torsional mode shapes were large. These results were consistent with the tendency for deflection and torsional deformation observed in Figure 11. In particular, the natural frequency was generally high when the LP was reinforced with GPLs. Considering the Campbell diagram results in Figure 12, the reinforcement of the LP with GPLs was more effective than the reinforcement of other positions in terms of vibration safety, as in the cases of deflection and torsional deformation.

### 3.3. Cost–Benefit Analysis

The change caused by reinforcing the wind turbine blade with a GPL was analyzed with respect to economic feasibility. The results obtained above showed that the most similar mechanical characteristics to the blade with conventional fiberglass composites occurred when the GPL content was 152.3 kg. In this instance, the total mass of the GPL-based blade was 13,209 kg, which was 21.5% lower than the previous mass of 16,829 kg. Although the density of the matrix (epoxy) of GPLRCs is similar to that of GPLs, as shown in Table 1, achieving a dramatic mass reduction effect simply by increasing the GPL content is difficult. Therefore, the mass should be effectively reduced by decreasing the thickness of the GPLRCs.

Hence, in this study, changes in the mechanical performance and mass of the wind turbine blade were analyzed while decreasing the thickness of the GPLRC and increasing the GPL content to achieve similar mechanical performance to the blades based on conventional fiberglass composites. Table 5 compares the maximum flapwise deflection, edgewise deflection, and torsional deformation of each case. Here, t is the modified thickness of the GPLRC, and $t_{o}$ is the thickness of the GPLRC that is identical to the thickness of the conventional fiberglass composites. The results of each case were obtained through repeated calculations to minimize the average errors from the maximum flapwise deflection, edgewise deflection, and torsional deformation obtained from the wind turbine blade with conventional fiberglass composites. Consequently, the value of $V_{GPL}^{*}$ gradually increased as the thickness of GPLRC decreased, and the performance for the maximum flapwise deflection and torsional deformation gradually improved. However, the maximum edgewise deflection remained similar to that of the conventional wind turbine blade regardless of the reduction in the GPLRC thickness.

Next, Table 6 contains the mass of each material and the total mass of the wind turbine blade according to the thickness ratio of the GPLRC. The total mass decreased by 21.5% to 52.1% compared to the wind turbine blade with fiberglass composites as the thickness of the GPLRC changed. In contrast, the mass of GPLs increased continuously to achieve similar mechanical performance to the conventional wind turbine blade, and it increased by up to 95.4% compared with $t/t_{o}=1.0$.

Finally, the economic feasibility of GPL-reinforced wind turbine blades was analyzed. Before the analysis, the costs of each material and the GPLs used in the wind turbine blades were investigated, and the results are presented in Table 7. The cost of GPLs was determined based on the data from CTI Materials [42]. For a conservative evaluation, the cost of the GPLs was assumed to be research-grade level, implying a relatively higher cost. For the other materials, the costs presented in the report by Bortolotti et al. [43] were referred to. Economic feasibility was analyzed based on the results in Table 6 and Table 7.

**Figure 14:**
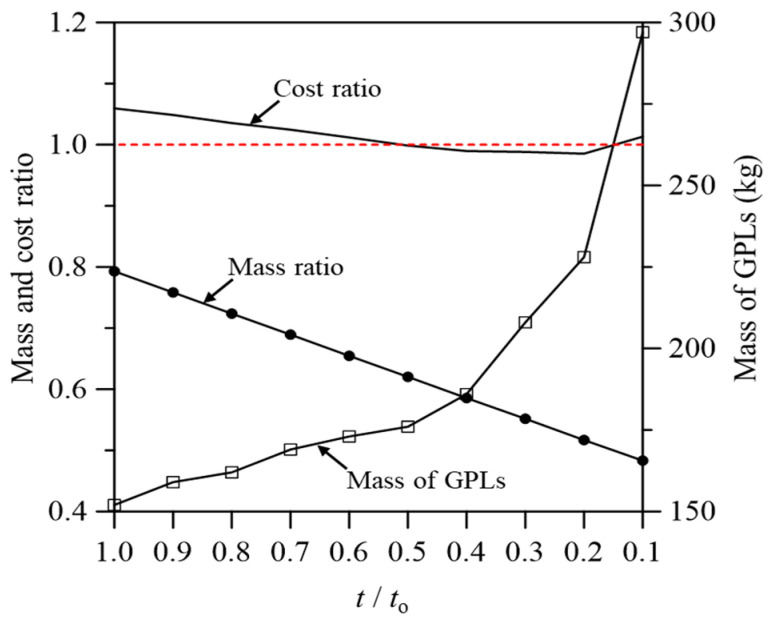
Variation of mass ratio, cost ratio, and mass of GPLs depending on the thickness ratio of the GPLRC.

Figure 14 shows the mass of GPLs, total mass, and the cost of the wind turbine blades according to the thickness ratio of the GPLRC. Here, the mass ratio and cost ratio indicate the ratios of the mass and cost of the GPLRC-based blade to those of the fiberglass-based blade. As in the results presented above, the mass of the GPLs increased as the thickness of the GPLRC decreased, but the total mass of the blade gradually decreased. In contrast, in terms of the material cost, the cost difference between the GPL-reinforced and unreinforced turbines was minimal (less than 10%). Even when $t/t_{o}$ was between 0.4 and 0.2, the material cost was calculated to be lower for the GPL-reinforced case. These findings indicate that using GPLRCs in place of fiberglass composites for wind turbine blades can significantly reduce the weight of the blades while keeping the material costs comparable.

## 4. Conclusions

In the present study, the mechanical performances and economic effects of graphene platelet (GPL)-reinforced wind turbine blades were analyzed to examine the applicability of GPLs as a future material. The reliability of the analysis results presented in this paper was verified by comparing the characteristics of the analysis model, such as mass, deflection, and torsion, with those of previous studies. In this instance, the 5MW-class SNL 61.5 m blade was used as the target model, and the aerodynamic loads acting on the blade were calculated using the blade element momentum theory (BEMT). The verified analysis model was used to analyze the mechanical performances (e.g., deflection, torsion, and natural frequency) of the wind turbine blade according to the GPL content and reinforcement position and the differences in the blade based on conventional fiberglass composites. In addition, economic feasibility was analyzed by investigating changes in mass and cost under the application of the GPL. The results of this study are summarized as follows:

- Flapwise deflection, edgewise deflection, and von Mises stress exhibited similar performance to the wind turbine blade based on conventional fiberglass composites when $V_{CNT}^{*}$ was 2.7%, whereas torsional deformation exhibited the most similar performance when $V_{CNT}^{*}$ was 2.0%;
- When the GPL reinforcement position (leading part (LP), trailing part (TP), spar cap, and shear web) in the wind turbine blade was different, fiberglass showed the largest flapwise deflection, succeeded by the TP, shear web, LP, and spar cap. The spar cap exhibited the largest edgewise deflection, succeeded by the fiberglass, shear web, LP, and TP. In addition, fiberglass had the largest torsional deformation, succeeded by the spar cap, TP, shear web, and LP;
- When the resonance of the analysis model was evaluated using a Campbell diagram, the possibility of resonance was lower for the blade with the GPLRC than that with fiberglass;
- When natural frequency characteristics were analyzed according to the GPL reinforcement position in the wind turbine blade, the same mode shape as the wind turbine blade with conventional fiberglass composites was observed in all cases. This study confirmed that the natural frequencies of the blades with the GPLRC were always higher than those with fiberglass;
- Similar mechanical performance was achieved by increasing the volume fraction of GPLs while significantly reducing the weight of the blade even if the thickness of the GPLRC in the wind turbine blade was reduced;
- When changes in the GPL content, blade weight, and cost due to the GPLRC thickness reduction were analyzed, the GPL content increased and the blade weight decreased as the thickness of the GPLRC decreased. The material cost remained similar to the blade based on conventional fiberglass composites.

Based on the outcomes of this study, GPL-reinforced wind turbine blades are expected to achieve high mechanical performance and have an economic effect by improving power generation efficiency and reducing the construction cost of wind turbine support structures through a reduction in the weight of the wind turbine blade. In addition, the cost of GPLs is gradually decreasing as a result of the increased production and the development of manufacturing technologies. The material cost is also expected to be significantly reduced by minimizing the amount of GPLs used in wind turbine blades through optimization. Furthermore, the manufacturing process of GPL-reinforced wind turbine blades is anticipated to be more straightforward than that of conventional fiberglass composites, as GPLRCs are a homogeneous and isotropic material, which eliminates the need for complicated lamination processes.

## Figures and Tables

**Table 1:** Material properties of GPL and epoxy [30].

Material	E (GPa)	$\nu_{12}$	ρ (kg/m^3^)
GPL	1010.0	0.186	1060
Epoxy	3.0	0.340	1200

**Table 2:** The computed effective material properties of the GPLRC.

$V_{GPL}$	$E_{eff}$ (GPa)	$\nu_{eff}$	$\rho_{eff}$ (kg/m^3^)
0.01 (1%)	11.8	0.338	1199
0.02 (2%)	20.7	0.337	1197
0.03 (3%)	30.0	0.335	1196
0.04 (4%)	38.5	0.334	1194

**Table 3:** Material masses in the SNL 61.5 m blade.

Model	Mass (kg)						
	Gelcoat	E-LT-5500 (UD)	Saertex (DB)	SNL (Triax)	Foam	Carbon (UD)	Total
Present	29	338	921	8726	4160	2655	16,829
Ref. [35]	29	376	916	8784	3953	2638	16,696

**Table 4:** Comparative analysis of the natural frequencies for the SNL 61.5m blade.

Model	Natural Frequency (Hz)					
	1st Flapwise	1st Edgewise	2nd Flapwise	2nd Edgewise	3rd Flapwise	1st Torsion
Present	0.8415	0.9930	2.7269	3.5918	5.7255	6.7280
Ref. [25]	0.87	1.06	2.68	3.91	5.57	6.45
Ref. [36]	0.90	-	2.85	-	6.41	6.65
Ref. [37]	0.9194	1.0552	2.8106	3.8870	5.6904	6.7152
Ref. [38]	0.84	0.969	2.41	-	-	-

**Table 5:** Variation of $V_{GPL}^{*}$, maximum deflections, and maximum torsional deformation in the wind turbine blade depending on the thickness ratio of the GPLRC.

Outcome	Fiberglass	$\mathrm{Thickness}\;\mathrm{ratio}\;\mathrm{of}\;\mathrm{GPLRC}\;(t/t_{o}$)									
		1.0	0.9	0.8	0.7	0.6	0.5	0.4	0.3	0.2	0.1
$V_{GPL}^{*}$ (%)	-	2.7	3.1	3.5	4.1	4.8	5.7	7.2	10.0	14.6	28.1
Max. flapwise deflection (m)	4.25	4.26	4.22	4.22	4.19	4.19	4.18	4.15	4.08	4.03	3.85
Max. edgewise deflection (m)	0.78	0.80	0.78	0.79	0.78	0.78	0.80	0.80	0.76	0.78	0.79
Max. torsional deformation (°)	−2.95	−2.31	−2.26	−2.26	−2.22	−2.20	−2.20	−2.14	−1.85	−1.94	−1.75

**Table 6:** Changes in the mass of materials and wind turbine blades depending on the thickness ratio of the GPLRC.

Material	Mass (kg)										
	Fiberglass	$\mathrm{Thickness}\;\mathrm{ratio}\;\mathrm{of}\;\mathrm{GPLRC}\;(t/t_{o}$)									
		1.0	0.9	0.8	0.7	0.6	0.5	0.4	0.3	0.2	0.1
Gelcoat	29	29	29	29	29	29	29	29	29	29	29
E-LT-5500	338	-	-	-	-	-	-	-	-	-	-
Saertex (DB)	921	-	-	-	-	-	-	-	-	-	-
SNL (Triax)	8726	-	-	-	-	-	-	-	-	-	-
Foam	4160	4160	4160	4160	4160	4160	4160	4160	4160	4160	4160
Carbon (UD)	2655	2655	2655	2655	2655	2655	2655	2655	2655	2655	2655
Epoxy	-	6213	5630	5050	4466	3885	3304	2717	2116	1512	861
GPL	-	152	159	162	169	173	176	186	208	228	297
Total weight	16,829	13,209	12,633	12,056	11,484	10,905	10,333	9755	9191	8611	8050

**Table 7:** Material costs of wind turbine blades [42,43].

Material	Cost (USD/kg)
Gelcoat	7.23
E-LT-5500(UD)	1.87
Saertex(DB)	3.00
SNL(Triax)	2.86
Foam	7.23
Carbon(UD)	30.00
Epoxy	3.63
GPL	90.00

## Data Availability

The original contributions presented in the study are included in the article, further inquiries can be directed to the corresponding author.

## Appendix A. Geometry and Material Properties of Wind Turbine Blades

**Table A1:** Geometric properties of the SNL 61.5 m wind turbine blade [25].

Blade Span (m)	Airfoil Type	Chord Length *L*_C_ (m)	Twist Angle (°)	Aero. Center *x*/*L*_C_
0	Circular	3.39	13.31	0.50
1.3667	Circular	3.39	13.31	0.50
10.25	DU40_A17	4.56	13.31	0.28
14.35	DU35_A17	4.65	11.48	0.28
22.55	DU30_A17	4.25	9.01	0.28
26.65	DU25_A17	4.01	7.80	0.28
30.75	DU25_A17	3.75	6.54	0.28
34.85	DU21_A17	3.50	5.36	0.28
38.95	DU21_A17	3.26	4.19	0.28
43.05	NA64_17	3.01	3.13	0.28
47.15	NA64_17	2.76	2.32	0.28
51.25	NA64_17	2.52	1.53	0.28
54.6667	NA64_17	2.31	0.86	0.28
57.4	NA64_17	2.09	0.37	0.28
60.1333	NA64_17	1.42	0.11	0.28
61.5	NA64_17	1.09	0.00	0.28

**Table A2:** Material properties of laminates [25].

Material	$E_{11}$ (GPa)	$E_{22}$ (GPa)	$G_{12}$ (GPa)	$\nu_{12}$	ρ (kg/m^3^)
Gelcoat	3.44	-	1.38	0.3	1235
E-LT-5500 (UD)	41.8	14.0	2.63	0.28	1920
Saertex (DB)	13.6	13.3	11.8	0.49	1780
SNL (Triax)	27.7	13.65	7.2	0.39	1850
Foam	0.256	0.256	0.022	0.3	200
Carbon (UD)	114.5	8.39	5.99	0.27	1220

**Table A3:** Stack IDs, names, and materials [25].

Stack ID	Stack Name	Material
1	Gelcoat	Gelcoat
2	Triax Skins	SNL (Triax)
3	Triax Root	SNL (Triax)
4	UD Carbon	Carbon (UD)
5	UD Glass TE	E-LT-5500 (UD)
6	TE Foam	Foam
7	LE Foam	Foam
8	Shear web facesheet	Saertex (DB)
9	Shear web core	Foam

**Table A4:** Stacking sequence in each panel of the blade model along the span [25].

Blade Span (m)	LE	LE Panel	Spar Cap	TE	TE Reinforcement	TE Panel	Shear Web
0	1-2-3-2	1-2-3-2	1-2-3-2	1-2-3-2	1-2-3-2	1-2-3-2	-
1.3667	1-2-3-2	1-2-3-2	1-2-3-2	1-2-3-2	1-2-3-2	1-2-3-2	8-9-8
1.5	1-2-3-2	1-2-3-7-2	1-2-3-4-2	1-2-3-2	1-2-3-5-6-2	1-2-3-6-2	8-9-8
6.8333	1-2-3-2	1-2-3-7-2	1-2-3-4-2	1-2-3-2	1-2-3-5-6-2	1-2-3-6-2	8-9-8
9	1-2-2	1-2-7-2	1-2-4-2	1-2-2	1-2-5-6-2	1-2-6-2	8-9-8
43.05	1-2-2	1-2-7-2	1-2-4-2	1-2-2	1-2-5-6-2	1-2-6-2	8-9-8
45	1-2-2	1-2-7-2	1-2-4-2	1-2-2	-	1-2-6-2	8-9-8
61.5	1-2-2	1-2-2	1-2-2	1-2-2	-	1-2-2	8-9-8

## Appendix B. Aerodynamic Load Calculation Using BEMT

Referring to Figure 2, showing the procedure of aerodynamic load calculation based on BEMT, the initial values of the axial and angular induction factors $a_{0}$ and $a\prime_{0}$ are set to zero. Next, the inflow angle φ and angle of attack α are calculated using Equations (A1) and (A2). The lift coefficient $C_{L}$, drag coefficient $C_{D}$, and pitching moment coefficient $C_{M}$ are determined corresponding to α, and the aerodynamic coefficient data of the SNL 61.5 m blade model are available in [44].

(A1) $$\tan\varphi =\frac{(1-a)V_{0}}{(1+a\prime )\Omega r}$$

(A2) α=φ−θ

where $V_{0}$ is the wind speed (11.4 m/s), r and Ω are the location in the blade length direction and the rotor’s angular velocity, and θ is the airfoil’s twist angle, respectively.

Once the aerodynamic coefficients are determined through the above procedure, the axial and angular factors $a_{k}$ and $a\prime_{k}$ can be updated through Equation (A3). This process should be iterated until the convergence tolerance ε is satisfied as defined in Equation (A4). In this study, 0.001 was used for the convergence tolerance ε.

(A3) $$a=\frac{1}{\frac{4\sin^{2}\varphi }{\sigma (C_{L}\cos\varphi +C_{D}\sin\varphi )}+1}\;,\;a\prime =\frac{1}{\frac{4\sin\varphi \cos\varphi }{\sigma (C_{L}\sin\varphi -C_{D}\cos\varphi )}-1}$$

(A4) $$\left|a_{k}-a_{k-1}\right|<\;\varepsilon \;\mathrm{and}\;\left|a\;\prime_{k}-a\;\prime_{k-1}\right|<\varepsilon$$

where σ=Bc/2πr, B is the number of blades in the wind turbine, and c is the chord length, respectively.

Finally, the lift force L, drag force D, and pitching moment M acting on the blade elements can be calculated through Equations (A5)–(A7) with the determined aerodynamic coefficients. The normal force $F_{N}$ and tangential force $F_{T}$ can then be derived from L and D as defined in Equations (A8) and (A9).

(A5) $$L=0.5\rho cV_{\mathrm{rel}}^{2}C_{L}dr$$

(A6) $$D=0.5\rho cV_{\mathrm{rel}}^{2}C_{D}dr$$

(A7) $$M=0.5\rho c^{2}V_{\mathrm{rel}}^{2}C_{M}dr$$

(A8) $$F_{N}=L\sin\varphi -D\cos\varphi$$

(A9) $$F_{T}=L\cos\varphi +D\sin\varphi$$

where ρ is the density of air, and the relative velocity is calculated as $V_{\mathrm{rel}}=\sqrt{\;{\left[(1-a)V_{0}\right]}^{2}+{\left[(1+a\prime )\Omega r\right]}^{2}}$.

## References

[1] Yee K., Ghayesh M.H. (2023). A review on the mechanics of graphene nanoplatelets reinforced structures. Int. J. Eng. Sci..

[2] Su X., Wang R., Li X., Araby S., Kuan H.C., Naeem M., Ma J. (2022). A comparative study of polymer nanocomposites containing multi-walled carbon nanotubes and graphene nanoplatelets. Nano Mater. Sci..

[3] Rafiee M.A., Rafiee J., Wang Z., Song H., Yu Z.Z., Koratkar N. (2009). Enhanced mechanical properties of nanocomposites at low graphene content. ACS Nano..

[4] Rafiee M.A., Rafiee J., Yu Z.Z., Koratkar N. (2009). Buckling resistant graphene nanocomposites. Appl. Phys. Lett..

[5] Parashar A., Mertiny P. (2012). Representative volume element to estimate buckling behavior of graphene/polymer nanocomposite. Nanoscale Res. Lett..

[6] Feng C., Kitipornchai S., Yang J. (2017). Nonlinear bending of polymer nanocomposite beams reinforced with non-uniformly distributed graphene platelets (GPLs). Compos. Part B Eng..

[7] Gholami R., Ansari R. (2017). Large deflection geometrically nonlinear analysis of functionally graded multilayer graphene platelet-reinforced polymer composite rectangular plates. Compos. Struct..

[8] Shi G., Araby S., Gibson C.T., Meng Q., Zhu S., Ma J. (2018). Graphene platelets and their polymer composites: Fabrication, structure, properties, and applications. Adv. Funct. Mater..

[9] Zhao S., Zhao Z., Yang Z., Ke L., Kitipornchai S., Yang J. (2020). Functionally graded graphene reinforced composite structures: A review. Eng. Struct..

[10] Mohan V.B., Lau K., Hui D., Bhattacharyya D. (2018). Graphene-based materials and their composites: A review on production, applications and product limitations. Compos. Part B Eng..

[11] UNFCCC (2015). Adoption of the Paris Agreement. FCCC/CP/2015/L.9/Rev.1.

[12] IMO (2023). 2023 IMO Strategy on Reduction of GHG Emissions from Ships.

[13] Mengal A.N., Karuppanan S., Wahab A.A. (2014). Basalt carbon hybrid composite for wind turbine rotor blades: A short review. Adv. Mat. Res..

[14] Chikhradze N.M. (2015). Hybrid fiber and nanopowder reinforced composites for wind turbine blades. J. Mat. Res. Technol..

[15] Muhammed K.A., Kannan C.R., Stalin B. (2020). Performance analysis of wind turbine blade materials using nanocomposites. Mater. Today Proc..

[16] Holmes J.W., Brøndsted P., Sørensen B.F., Jiang Z., Sun Z., Chen X. (2009). Development of a bamboo-based composite as a sustainable green material for wind turbine blades. Wind. Eng..

[17] Shen-xue J., Qi-sheng Z., Shu-hai J. (2002). On Structure, production, and market of bamboo-based panels in China. J. Forestry Res..

[18] Ennis B.L., Kelley C.L., Naughton B.T., Norris R.E., Das S., Lee D., Miller D.A. (2019). Optimized Carbon Fiber Composites in Wind Turbine Blade Design.

[19] Paquette J., van Dam J., Hughes S. Structural testing of 9m carbon fiber wind turbine research blades. Proceedings of the AIAA 2007-816 45th AIAA Aerospace Sciences Meeting and Exhibit.

[20] Boncel S., Kolanowska A., Kuziel A.W., Krzyżewska I. (2018). Carbon nanotube wind turbine blades: How far are we today fromlaboratory tests to industrial implementation. ACS Appl. Nano. Mater..

[21] Buyuknalcaci F.N., Polat Y., Negawo T.A., Döner E., Alam M.S., Hamouda T., Kilic A. (2018). 24—Carbon nanotube-based nanocomposites for wind turbine applications. Polymer-Based Nanocomposites for Energy and Environmental Applications.

[22] Saboori A., Pavese M., Badini C., Fino P. (2017). Effect of sample preparation on the microstructural evaluation of Al–GNPs nanocomposites. Metallogr. Microstruct. Anal..

[23] Shamsaei E., de Souza F.B., Yao X., Benhelal E., Akbari A., Duan W. (2018). Graphene-based nanosheets for stronger and more durable concrete: A review. Constr. Build. Mater..

[24] Scidà A., Haque S., Treossi E., Robinson A., Smerzi S., Ravesi S., Borini S., Palermo V. (2018). Application of graphene-based flexible antennas in consumer electronic devices. Mater. Today..

[25] Resor B.R. (2013). Definition of a 5MW/61.5m Wind Turbine Blade Reference Model.

[26] Wu H., Kitipornchai S., Yang J. (2017). Thermal buckling and postbuckling of functionally graded grapheme nanocomposite plates. Mater. Des..

[27] Yang Z., Liu A., Yang J., Lai S.-K., Lv J., Fu J. (2021). Analytical Prediction for Nonlinear Buckling of Elastically Supported FG-GPLRC Arches under a Central Point Load. Materials.

[28] Habibi M., Mohammadi A., Safarpour H., Ghadiri M. (2019). Effect of porosity on buckling and vibrational characteristics of the imperfect GPLRC composite nanoshell. Mech. Based Des. Struc..

[29] Javani M., Kiani Y., Eslami M.R. (2021). Geometrically nonlinear free vibration of FG-GPLRC circular plate on the nonlinear elastic foundation. Compos. Struct..

[30] Cho J.R. (2023). Free vibration analysis of functionally graded porous cylindrical panels reinforced with graphene platelets. Nanomater..

[31] Zhang C., Chen H.P., Huang T.L. (2018). Fatigue damage assessment of wind turbine composite blades using corrected blade element momentum theory. Measurement.

[32] Algolfat A., Wang W.A. (2022). Albarbar, Dynamic response analysis of a 5 MW NREL wind turbine blade under flap-wise and edge-wise vibrations. J. Dyna. Monitor. Diagn..

[33] Ullah H., Ullah B., Silberschmidt V.V. (2020). Structural integrity analysis and damage assessment of a long composite wind turbine blade under extreme loading. Compos. Struct..

[34] Sang S., Wen H., Cao A.X., Du X.R., Zhu X., Shi Q., Qiu C.H. (2020). Dynamic modification method for BEM of wind turbine considering the joint action of installation angle and structural pendulum motion. Ocean Eng..

[35] Chen Z.J., Stol K.A., Mace B.R. (2017). Wind turbine blade optimisation with individual pitch and trailingedge flap control. Renew. Energy.

[36] Shakya P., Sunny M.R., Maiti D.K. (2019). A parametric study of flutter behavior of a composite wind turbine blade with bend-twist coupling. Compos. Struct..

[37] Johnson E.L., Hsu M.C. (2020). Isogeometric analysis of ice accretion on wind turbine blades. Comput. Mech..

[38] Thapa M., Missoum S. (2022). Uncertainty quantification and global sensitivity analysis of composite wind turbine blades. Reliab. Eng. Syst. Safe..

[39] Jeong M.S., Cha M.C., Kim S.W., Lee I., Kim T. (2014). Effects of torsional degree of freedom, geometric nonlinearity, and gravity on aeroelastic behavior of largescale horizontal axis wind turbine blades under varying wind speed conditions. J. Renew. Sustain. Ener..

[40] Li Z., Wen B., Dong X., Peng Z., Qu Y., Zhang W. (2020). Aerodynamic and aeroelastic characteristics of flexible wind turbine blades under periodic unsteady inflows. J. Wind. Eng. Ind. Aerod..

[41] Yu D.O., Kwon O.J. A coupled CFD-CSD method for predicting HAWT rotor blade performance. Proceedings of the 51st AIAA Aerospace Sciences Meeting including the New Horizons Forum and Aerospace Exposition.

[42] CTI Materials. https://www.ctimaterials.com.

[43] Bortolotti P., Berry D., Murray R., Gaertner E., Jenne D., Damiani R., Barter G., Dykes K. (2019). A Detailed Wind Turbine Blade Cost Model.

[44] Kim H.J., Cho J.-R. (2024). Effects of graphene reinforcement on static bending, free vibration, and torsion of wind turbine blades. Materials.

